# U.S. primary care physician perceptions on barriers to providing guideline-driven care for UTI and recurrent UTI: a qualitative study

**DOI:** 10.1186/s12875-024-02477-3

**Published:** 2024-07-01

**Authors:** Jennifer Park, Michele Torosis, Ja-Hong Kim, A. Lenore Ackerman

**Affiliations:** 1 Department of Obstetrics and Gynecology, David Geffen School of Medicine, University of California, Box 951738, Los Angeles, CA 90095-1738 USA; 2 Department of Urology, Division of Pelvic Medicine and Reconstructive Surgery, David Geffen School of Medicine, University of California, Los Angeles, Los Angeles, CA 90095 USA

**Keywords:** Urinary tract infection, Antibiotic resistance, Recurrent UTI, UTI guidelines, Urine culture

## Abstract

**Background:**

Urinary tract infections (UTI) affect almost two-thirds of all women during their lives and many experience recurrent infections. There are evidence-based guidelines from multiple international societies for evaluation and treatment; however, recent claims-based analyses have demonstrated that adherence to these guidelines is poor. This study seeks to understand the barriers experienced by U.S. primary care providers (PCPs) to providing guideline-based care for UTI and recurrent UTI (rUTI).

**Methods:**

Semi-structured interviews of 18 PCPs, recruited from the greater Los Angeles area, examined real-world clinical management of UTI/rUTI episodes, decisions to refer to subspecialty care, and resources guiding counseling and management. Grounded theory methodology served to analyze interview transcripts and identify preliminary and major themes.

**Results:**

Participants expressed the desire to obtain urine cultures for each cystitis episode, but felt pressured to make compromises by patient demands or barriers to care. PCPs had lower thresholds to empirical treatment if patients had a history of rUTIs, were elderly, or declined evaluation. Laboratory data was minimally utilized in clinical decision-making: urinalyses were infrequently considered when interpreting culture data. PCPs treated a broad set of urologic and non-urologic symptoms as UTI, even with negative cultures. PCPs did not feel comfortable initiating UTI prophylaxis, instead seeking specialist evaluation for anatomic causes. They were unaware of management guidelines, typically utilizing UpToDate® as their primary resource. Few evidence-based UTI prevention interventions were recommended by providers.

**Conclusions:**

Low availability of succinct and clear professional guidelines are substantial barriers to appropriate UTI/rUTI care. Poor useability of clinical guidance documents results in substantial confusion about the role of preventative measures and additional diagnostic testing. Difficulties in patient access to care providers lead to expectations for presumptive treatment. Future studies are needed to determine if improved educational materials for providers and/or management algorithms can improve guideline concordance of UTI management.

## Background

Urinary tract infections (UTI) affect approximately 60% of all women throughout their lives, with 20–40% of women experiencing recurrent infection [1]. UTIs result in more than 8 million ambulatory visits each year, resulting in annual direct U.S. healthcare costs of $1.6 billion [2]. Evidence-based guidelines from multiple international societies, including the Infectious Diseases Society of America (IDSA) [3], American Urological Association (AUA) [1], American Academy of Family Physicians (AAFP) [4], and American Urogynecologic Society (AUGS) [5], have established pathways for the evaluation and management of these conditions with the goals of encouraging comprehensive diagnostic evaluation, individualized patient care, and antibiotic stewardship.

Numerous recent claims-based analyses, however, have demonstrated that adherence to these guidelines is poor [6–8]. Guidelines stress that urine studies (urinalysis and urine culture/susceptibility) are critical in diagnosis and management, and should be evaluated with each episode of symptomatic cystitis, yet less than 35% of visits for UTI are associated with urine culture testing [9]. In reviewing referral patterns for recurrent UTI (rUTI) from primary care physicians to urology, 49% did not have any positive urine cultures at the time of referral and 35% had no documentation of any cultures ever having been obtained, despite multiple antibiotic courses [10]. Guidelines also emphasize the importance of a complete history and physical examination, including pelvic exam in women, particularly in patients with recurrent infections. However, pelvic exams were performed in only 21% of patients with recurrent UTIs [1]. 

UTI is a leading cause for antibiotic prescriptions, second only to respiratory infections, but as many as 30–60% of these prescriptions may be inappropriate [6–8]. Guideline consensus, as well as guidance from the Centers for Disease Control and Prevention (CDC) [11], establish short courses of nitrofurantoin, sulfamethoxazole-trimethoprim, or fosfomycin as first-line agents in the treatment of UTI. However, only 34% of outpatient antibiotic prescriptions for cystitis are of an appropriate antibiotic at the correct dose and duration. Antibiotics are frequently prescribed for negative cultures or symptoms inconsistent with UTI [7, 12, 13]. 

Recurrent infections should prompt prophylactic measures, including oral hydration, prophylactic antimicrobial agents, vaginal estrogen, and avoidance of agents that increase UTI risk, such as spermicide or douching. The AAFP affirms that most patients with recurrent, uncomplicated UTIs can be treated by family physicians, including the institution of prophylactic measures [4]. Yet despite lower rates of UTI occurrence, reduced antibiotic burden, and decreases in multidrug resistant bacteria with prophylaxis, fewer than 1 in 7 patients are prescribed any regimen to prevent UTI. In a sampling of patients referred to urology for rUTI, none had received any form of prophylaxis prior to referral [1, 14]. 

While these data suggest poor adherence to current guidelines for UTI management, little is understood of the barriers to providing guidelines-based care facing primary care providers (PCPs). Our objective was to examine the factors driving primary care practice patterns for the management of UTI and rUTI. We sought to understand the clinical factors influencing current practice patterns and referrals to urologic specialty care and identify barriers in primary care rUTI management that may lead to inappropriate care.

## Methods

This qualitative interview-based study was exempt from institutional review board review by the University of California, Los Angeles Office of the Human Research Protection (IRB#21-001396).

Participants were recruited through the Internal and Family Medicine directories of UCLA Health, a single healthcare system serving over 700,000 patients. All primary care providers were contacted via email introducing the study and asking for interest in participation. Twelve interviews of a homogenous group is needed to reach saturation of concepts in grounded theory studies; [15] to ensure adequate representation of the range of practice environments. From interested physicians who responded to the recruitment email were contacted for study involvement, 20 were selected to reflect a diversity of locations, practice environments, and experience, of whom 18 completed the interviews.

Telephone interviews were conducted and digitally recorded by study personnel. A semi-structured interview template guided conversations using an interview template created by the interdisciplinary research team. Inquiry domains included diagnosis and evaluation of acute cystitis, treatment of first and recurrent cystitis episodes, counseling regarding UTI prevention, criteria for referral to specialists, and resources used to aid treatment of these patients (Table 1). Participants received a $50 gift card for participation. Interviews were transcribed verbatim by a transcription service (Temi, San Francisco, CA) using audio recordings of the call.

**Table 1:** Semi-structured interview sample questions

Topics	Sample Questions
UTI diagnosis	- What are the symptoms of a UTI? - When a patient calls your office or sends you a message regarding UTI symptoms, what is your next step? How often do you treat empirically over the phone vs. request the patient to submit a urine sample at a lab vs. request them to schedule a clinic visit? - At what point in your work up do you obtain a urine culture? - Do you perform a pelvic exam for patients with UTI complaints? - Do you always obtain a UA with your urine cultures? - Does the UA change your management?
UTI Treatment	- Do you empirically treat new onset UTI symptoms in patients without a history of UTIs? - For a patient with a positive urine culture, what are your first line antibiotics? - If you treat a patient, and they subsequently do not symptomatically improve after the first course of antibiotics, what is your next step in management?
Referral for rUTI	- If you make a referral for rUTI, what do you counsel patients regarding this? - When you make these referrals, do you feel that it is for rUTI or to evaluate for another possible etiology? - When do you decide to refer to urology or urogynecology for rUTIs? What criteria do you use? - What work up do you think the consulting provider needs in order to evaluate the patient?
Prevention and counseling	- Do you recommend patients to start any UTI prevention strategies? If so, which interventions do you recommend? - Where do you obtain these recommendations? What are your resources?

Data were analyzed using grounded theory methodology [16], a structured methodology to study the experience of participants, particularly when little is known about a phenomenon. Grounded theory allows organization of collected qualitative data into codes that are subsequently formed into recurring themes amongst all responses and finally into an inductively resultant theory. Interview transcripts were coded independently by two research team members, who arrived at recurring themes individually. After double coding was performed, the research team met to discuss themes, assess for thematic saturation, and identify core categories. Dedoose software, version 8.3.17 (Los Angeles, CA), facilitated data management and coding.

## Results

Seventeen physicians and one nurse practitioner (9 male, 9 female) with a median of 11 years of clinical experience (range 4–34) were interviewed. 61% of the 18 providers worked at community-based clinics, while 39% worked in hospital-adjacent facilities. Participants serve a socioeconomically diverse patient population across metropolitan Los Angeles County.

Thematic saturation was met and consensus was achieved on six key themes surrounding the evaluation and management of cystitis in the primary care setting, for which representative quotes are included by theme (Table 2). Primary care physicians expressed a desire to follow guidelines and obtain culture data for patients with cystitis symptoms, however, they felt they were limited by patients’ expectations for care. Symptoms were the main driver of antibiotic treatment and carried more weight than culture data or exam findings. PCPs were hesitant to move forward with treating rUTIs without anatomic evaluation and expressed a desire for more educational resources on UTI prevention.

**Table 2:** Themes and illustrative quotes pertaining to themes

**Themes related to management/prevention of UTIs**	**Quotes**
Patient expectations dictating antibiotic use	“If I’m not able to get them to come in, I will usually ask them to get urine collected…there’s a lot of bargaining there…If they’ve convinced me that their barriers are super high, sometimes I’ll do the lesser of two evils, just send them in a few days of Bactrim.” “I can’t remember the last time I was able to convince a person, a young woman to come into the office for those symptoms.”
Symptoms guiding treatment over laboratory findings	“If the urine dipstick was negative, I would allow that to be a shared decision making. I would tell the patient it’s less likely to be a UTI given the fact that it’s completely negative, but we can treat it or we can wait for the culture just to make sure.” “In fact, I would place more weight on the history than I would on a urinalysis in some circumstances.” “If they’re older, had recurrent UTIs before - there’s somebody who if we missed a UTI next week, they could be in urosepsis, because they’re so old and fragile, then I’ll go ahead and start it.“ “So, you know, a young woman who’s had a previous UTI, she comes in because she went with her partner to Las Vegas. They had a lot of sexual intercourse, now she’s got her typical symptoms, you know, that patient, I probably just treat empirically.”
De-emphasis of the importance of physical exam	“For UTIs, I don’t do pelvic exams unless the UA is bland and there some concern for vaginal issues, but if it’s the UA is consistent with UTI and this history is as well, I’ll just treat empirically. I don’t even do really any exam.” “I probably never [do exams for UTI patients]. I probably wouldn’t know what I would be looking for and that’s like, never crossed my mind to do” “I typically do not do pelvic exam unless they’re having vaginal symptoms.”
Desire for more recommendations surrounding UTI care	“Just over the years, or residency or seeing patients and gosh, I mean I don’t know I hope it’s somewhat based on something from evidence at some point that I heard.” “I recommend cleaning from front to back, not from back to front, just in case it is *E. Coli* causing the UTI…We also recommend drinking cranberry juice.” “I will look at UpToDate periodically to see if there’s been a change in any of the guidelines as it relates to antibiotics.” “I just use what I learned in residency, but I would love more resources. I definitely don’t think we got enough training.”
**Themes related to referral to subspecialist for rUTIs**	**Quotes**
Lack of consensus on in specialist referral	“So there is not like a hard stop number. It sort of just depends on how frequently they’re coming in. But if… they’re in my office month after month after month after month and we’ve tried all kinds of stuff and nothing is working and I suspect there’s… I don’t know, let’s say it’s anatomical or something, then I would probably err on the side of sending the referral because I’m sort of running out of options myself.” “If the patient doesn’t want to see a lot of doctors and it’s not bothering them, I probably might have a higher threshold to send them. But if there’s someone who’s very worried about it, I tend to give into that and probably refer them a lot sooner.”
Fear of missing underlying anatomic abnormality	“I just want urology to make sure there is no malignancy or something rare like TB. And make sure they are emptying their bladder all the way, etc.” “I would need their recommendations from them to continue treating the patients. I would tell them [the patient] to expect further workup as far as imaging or procedures or things like that.” “When there’s rUTIs and that’s when I worry about something structural, you know, is there a stone, is there a something going on that’s serving as a nidus of infection, is there a problem with your emptying or filling or relaxation? And at that point then that’s, that’s when I encourage and refer to urology to do the urodynamic flow testing and any other sort of imaging workup that’s needed.”

### Patient expectations dictating antibiotic use

PCPs expressed an understanding of the utility of and a desire to obtain urine culture information for all patients upon each presentation for UTI symptoms. They felt pressured, however, to make compromises in this standard because of patient demands or barriers to care. PCPs reported pressure from patients to prescribe antibiotics immediately, without urine laboratory evaluation or clinic encounter, as this was viewed as an inconvenience and barrier to the desired care. There was greater fear of the risk of a missed or untreated infection, compared with the risk of inappropriate or unnecessary antibiotic use. If a patient had previously had one positive culture or was young and sexually active, PCPs were less likely to push for repeat cultures with subsequent events. More liberal, empiric treatment of older patients was ascribed to the belief that empiric treatment would prevent infectious progression to bacteremia, particularly in frail individuals.

### Symptoms guiding treatment over laboratory findings

If a urinalysis was negative (no leukocytes or bacteria), but symptoms were consistent with cystitis, primary care physicians felt comfortable treating the patient with antibiotics based on symptoms alone, particularly if this was the first presentation of such symptoms. Any of the following were considered to be cystitis symptoms by most PCPs surveyed: dysuria, frequency, urgency, fever, foul smelling urine, cloudy urine, incontinence, and changes in mental status. Urine cultures were still sent in the setting of negative urinalysis; none of the PCPs considered bacterial colonization, asymptomatic bacteriuria, or specimen contamination as a possible explanation for negative urinalysis with positive culture. In this scenario, patients whose symptoms persisted despite antibiotic use were either offered repeat laboratory testing or a second or extended antibiotic course.

### Desire for more recommendations surrounding UTI care

PCPs uniformly expressed a lack of appropriate guidance on how to manage rUTI. When asked which guidelines they referenced to determine next steps for UTI care, UpToDate® emerged as the primary resource. Many providers drew upon recollections from their training, and used patient information embedded in the electronic medical record for patient education on UTI prevention. PCPs recommended to patients to wipe from front to back, post-coital voiding, increasing oral hydration, and cranberry supplements. PCPs frequently expressed the desire to broaden their knowledge base and voiced a need for more available resources. Few PCPs were aware of the AAFP guidelines for rUTI treatment, and none cited addition guidelines, such as those from IDSA or AUA, as reference tools to manage recurrent infections.

### De-emphasis of the importance of physical exam

For the subset of patients that were able to present to the office, only 31% of providers routinely performed pelvic exams when evaluating a possible UTI, despite 78% of providers stating they felt comfortable performing a pelvic exam. PCPs who did not routinely perform pelvic exams for UTI symptoms stated they were not aware it was needed or did not know what to look for on pelvic exam for this indication. A subset stated they would only perform an exam if vaginal-specific symptoms were expressed or if the urine dipstick was negative. PCPs voiced that the ability to do a urine dipstick analysis drove them to bring patients in for UTI symptoms more than the need for physical exam.

### Lack of consensus on specialist referral

The number of UTI episodes prompting specialist referral varied widely between providers. Providers cited between 3 and 6 UTIs in a year as a trigger for referral, with a lower threshold in elderly patients, those who requested referrals, or individuals with medical comorbidities or persistent infections despite behavioral modifications.

### Fear of missing underlying anatomic abnormality

PCPs cited the desire for anatomic evaluation as the main reason for referral to a specialist for evaluation. In rUTI patients, PCPs expressed a fear of missing anatomic abnormalities that increase rUTI risk. Almost uniformly, PCPs anticipated patients would receive further workup at the subspecialist’s office with cystoscopy and imaging. Many providers preemptively ordered imaging, most commonly renal ultrasound or CT, to facilitate subspecialist diagnostic work up. PCPs rarely felt comfortable initiating UTI prophylaxis without an anatomic evaluation and recommendations from a specialist. PCPs were aware of measures such as vaginal estrogen, methenamine hippurate, D-mannose, and post-coital antibiotics, but did not feel it was within their scope of practice to initiate these measures.

## Discussion

Poor adherence to clinical guidelines is a common problem affecting a wide range of medical issues, including cardiovascular care, diabetes, and cancer screening [17]. Despite availability of clinical practice guidelines by multiple international societies addressing the diagnosis and management of UTI and rUTI, PCPs often diverge from these care principles. While individual guidelines differ on some of the details regarding diagnosis and management of rUTI and acute cystitis, these variations in guideline position did not appear to influence the variability in care delivery patterns. Instead, PCPs enumerated individual and institutional obstacles to practicing evidence-based, guideline-driven care of UTI, including a desire to maintain patient satisfaction, poor familiarity with and/or misinterpretation of the guidelines, and low availability of accurate practice guidance materials for physicians. These observations align well with barriers to providing evidence-based care in other health care systems outside the U.S [18, 19]. 

As supported by multiple guidelines, patients should be evaluated with history and physical exam, including pelvic exam, as multiple confounding pathologies such as vaginitis, sexually transmitted infections, vulvar dermatoses, and overactive bladder can present with cystitis-like symptoms. Our data, however, reveal that PCPs did not consider physical exam, specifically pelvic exam, a critical tool in UTI diagnosis. Providers were unsure of what to evaluate on pelvic exam and how this could identify confounding conditions. The combination of sudden-onset dysuria and frequency without vaginal discharge confers a 90% probability of UTI, leading some to believe physical exam is not necessary [20]. Without that specific combination of symptoms, however, ~ 50% of women with one or more UTI symptoms (dysuria, frequency, urgency, hematuria, or back pain) did not have cystitis, suggesting misdiagnosis is common [20]. In person assessment significantly improves UTI diagnostic accuracy; culture positivity rates for those tested following virtual assessment (29%) are less than half that for patients seen in person (64%), despite similar frequencies of obtaining urine studies [9]. 

Consensus among guidelines supports that diagnosis of UTI requires specific cystitis symptoms as well as objective confirmation of UTI diagnosis with urine testing. Several symptoms widely considered by PCPs to be indicative of UTI, such as foul-smelling urine or change in mental status, are not true cystitis symptoms; multiple guidelines document that urine testing is not indicated for these symptoms alone. Each episode of cystitis should be treated as a discrete event; confirmation of the offending microbe and antibiotic susceptibility patterns can guide clinicians in determining treatment and prevention strategies [1]. Most PCPs asserted agreement with this recommendation, but felt patient expectations for presumptive treatment constituted a significant obstacle. Particularly in patients with prior infections, providers experienced substantial patient opposition to present for in-person or laboratory assessment. However, with shared decision marking reviewing antibiotic risks, the rarity of infection progression, and similar rates of resolution without antibiotics, the majority of patients are willing to forgo early antibiotic treatment [21–23]. Combined with recent data noting significant patient fear of repeated antibiotic treatments in rUTI [24], this obstacle may be overcome with improved patient/provider discussion with the assistance of patient decision aids and advance planning for acute episodes [25, 26]. 

In the setting of negative urinalysis or urine culture, many providers felt antibiotic treatment was still reasonable, despite a high negative predictive value of urine dipstick in UTI diagnosis [27]. PCPs were more likely to empirically prescribe antibiotics regardless of laboratory findings in certain populations, such as sexually active women. Even without urinary symptoms, PCPs reported a lower threshold to treat geriatric patients with antibiotics for a positive culture, citing concern for progression to bacteremia. Ignoring positive urinalysis or culture for patients with vague symptoms or chronic urinary issues made providers uneasy; the perception that untreated asymptomatic bacteriuria is dangerous persists as a major barrier to antibiotic stewardship [28]. 

This overall attitude indicates several provider beliefs about antibiotics: (1) that the risks of antibiotics, even if inappropriate, are minimal (both in terms of individual adverse events and potential development of antimicrobial resistance) (2) antibiotics may have utility in patients with negative urine testing, and (3) that concern for more serious infections justifies broader use, especially in older adults. PCPs expressed a perceived greater risk in letting bacteriuria (symptomatic or asymptomatic) remain untreated than in the sequalae of antibiotic overuse, beliefs that are associated with poorer adherence to guideline-concordant care [29]. While these beliefs in a positive benefit-to-risk ratio for presumptive and empiric antibiotics persist, expectations that non-guideline-based antibiotic treatment can avert more serious infections, reduce UTI episodes, or increase the time between acute cystitis episodes have been disproven [30]. Numerous studies document that the negative effects of multiple antibiotics accumulate over time [31, 32]. In addition to worsening antibiotic resistance, individual consequences of repeated intermittent antibiotics can include allergic reactions, organ toxicities, *C. difficile* infections, and persistent post-antibiotic associated diarrhea [33]. Women receiving repeated antibiotics attempting to eradicate bacteriuria had higher frequencies of symptomatic cystitis [31], higher prevalence of antibiotic resistance, higher incidences of pyelonephritis, and reduced quality of life in comparison to women receiving placebo [32]. 

To avoid these consequences, both the IDSA and American Geriatric Society have specific recommendations guiding appropriate antibiotic use for treatment of bacteriuria, acknowledging high rates of benign bacteriuria in older adults and poor correlations between symptoms and cultures. Yet as no guideline offers definitive culture criteria or symptomatic requirements for UTI diagnosis, overtreatment is both understandable and difficult to correct [34]. 

Providers expressed limited comfort with the evaluation and management of rUTI. While several guidelines suggest referral to a specialist when an anatomic or functional contribution is suspected, few provide guidance on the patient features that should prompt a provider to suspect such causes. Responses from PCPs suggest their perception that abnormal anatomy plays a role in rUTI susceptibility in most women, as evidenced by the priority PCPs placed on ordering imaging to work-up rUTI. Multiple prospective trials, however, demonstrate that more than 99% of specialist evaluations (imaging and cystoscopy) do not reveal any underlying pathology [35]. In addition, most providers felt uncomfortable initiating preventative measures until such a work-up was completed, instead recommending behavioral measures (such as wiping front-to-back) that lack evidence for efficacy. In contrast, consensus recommendations reinforce the concept that antibiotic prophylaxis can be safely instituted before any work up; numerous randomized controlled trials document most patients will experience remissions in UTI with appropriate preventative measures [1, 36, 37]. Several treatment and preventative care algorithms exist to guide providers in rUTI prevention, including an UpToDate® overview guiding physicians on approaches to prophylactic therapy. Our PCP interviewees, however, both were unaware of the breadth of clinical guidance documents and had difficulty accessing these resources.

These data combine to reveal profound shortcomings in the available guidance concerning appropriate UTI/rUTI management. No consensus exists among available clinical guidelines on objective diagnostic criteria, expectations for preventative management, or indications for referral. This lack of consensus contributes to heterogeneous diagnostic, treatment, evaluation, and referral patterns among primary care providers, even within a single healthcare system. A single multidisciplinary guide may simplify management algorithms, clarify validated UTI symptoms, and give providers confidence to reserve antibiotics for when they are likely to impact outcomes. Lastly, electronic decision support tools could improve care in a multitude of ways, from recognizing patients with multiple cystitis episodes who would benefit from preventative management to digital order sets encouraging appropriate testing and choice of antibiotic type and duration. Such interventions supporting good clinical practice are both practical and possible, particularly with the framework of electronic medical systems.

This scientific survey of primary care providers is strengthened by the open question format, allowing a detailed exploration of the obstacles underlying the numerous reports documenting poor clinical management of rUTI. The PCPs interviewed reflected a broad range of practice settings serving an ethnically and socioeconomically diverse patient population across a wide geographical region. This study, however, is limited by the recruitment of PCPs from a single healthcare delivery system in a large metropolitan area. Additionally, we recognize the possibility of volunteer bias amongst the PCPs in this study. Thus, these practice patterns may not reflect care across the U.S.; providers in other types of communities may experience additional personal and systemic barriers to delivering appropriate UTI care. However, we anticipate that many of the barriers described here would be amplified in lower resource settings. Only by understanding barriers for primary care physicians in management of rUTI can we hope to implement scalable interventions to improve the care of this highly prevalent and frequently debilitating condition with better diagnostic accuracy, decreased healthcare costs, appropriate antibiotic stewardship, and individualized management.

## Conclusion

Despite widely available practice guidelines overviewing UTI diagnostic work-up and management, PCPs often deviate from standards of care. Patient expectations, poor useability of guidelines and low availability of accurate provider guidance on the management and treatment of UTI/rUTI were the greatest barriers to appropriate and complete care.

## Data Availability

The datasets used and/or analysed during the current study are available from the corresponding author on reasonable request.

## Abbreviations

UTI: Urinary tract infections
rUTI: Recurrent urinary tract infection
PCPs: Primary care providers
IDSA: Infectious Diseases Society of America
AUA: American Urological Association
AAFP: American Academy of Family Physicians
AUGS: American Urogynecologic Society
CDC: Centers for Disease Control and Prevention
